# Widespread Volumetric Brain Changes following Tooth Loss in Female Mice

**DOI:** 10.3389/fnana.2016.00121

**Published:** 2017-01-09

**Authors:** Limor Avivi-Arber, Ze'ev Seltzer, Miriam Friedel, Jason P. Lerch, Massieh Moayedi, Karen D. Davis, Barry J. Sessle

**Affiliations:** ^1^University of Toronto Centre for the Study of Pain, University of TorontoToronto, ON, Canada; ^2^Faculty of Dentistry, University of TorontoToronto, ON, Canada; ^3^Department of Physiology, Faculty of Medicine, University of TorontoToronto, ON, Canada; ^4^Department of Anesthesiology, University Health NetworkToronto, ON, Canada; ^5^Central Institute of Mental Health, University of HeidelbergMannheim, Germany; ^6^Mouse Imaging Centre, Hospital for Sick ChildrenToronto, ON, Canada; ^7^Medical Biophysics, Faculty of Medicine, University of TorontoToronto, ON, Canada; ^8^Department of Surgery, University of TorontoToronto, ON, Canada; ^9^Institute of Medical Science, University of TorontoToronto, ON, Canada; ^10^Division of Brain, Imaging and Behaviour - Systems Neuroscience, Krembil Research Institute, Toronto Western Hospital, University Health NetworkToronto, ON, Canada

**Keywords:** tooth loss, trigeminal nerve, sMRI, brain imaging (MRI), plasticity, neuroplasticity, genetic variation, animal model

## Abstract

Tooth loss is associated with altered sensory, motor, cognitive and emotional functions. These changes vary highly in the population and are accompanied by structural and functional changes in brain regions mediating these functions. It is unclear to what extent this variability in behavior and function is caused by genetic and/or environmental determinants and which brain regions undergo structural plasticity that mediates these changes. Thus, the overall goal of our research program is to identify genetic variants that control structural and functional plasticity following tooth loss. As a step toward this goal, here our aim was to determine whether structural magnetic resonance imaging (sMRI) is sensitive to detect quantifiable volumetric differences in the brains of mice of different genetic background receiving tooth extraction or sham operation. We used 67 adult female mice of 7 strains, comprising the A/J (A) and C57BL/6J (B) strains and a randomly selected sample of 5 of the 23 AXB-BXA strains (AXB1, AXB4, AXB24, BXA14, BXA24) that were produced from the A and B parental mice by recombinations and inbreeding. This panel of 25 inbred strains of genetically diverse inbred strains of mice is used for mapping chromosomal intervals throughout the genome that harbor candidate genes controlling the phenotypic variance of any trait under study. Under general anesthesia, 39 mice received extraction of 3 right maxillary molar teeth and 28 mice received sham operation. On post-extraction day 21, *post-mortem* whole-brain high-resolution sMRI was used to quantify the volume of 160 brain regions. Compared to sham operation, tooth extraction was associated with a significantly reduced regional and voxel-wise volumes of cortical brain regions involved in processing somatosensory, motor, cognitive and emotional functions, and increased volumes in subcortical sensorimotor and temporal limbic forebrain regions including the amygdala. Additionally, comparison of the 10 BXA14 and 21 BXA24 mice revealed significant volumetric differences between the two strains in several brain regions. These findings highlight the utility of high-resolution sMRI for studying tooth loss-induced structural brain plasticity in mice, and provide a foundation for further phenotyping structural brain changes following tooth loss in the full AXB-BXA panel to facilitate mapping genes that control brain plasticity following orofacial injury.

## Introduction

Loss of teeth remains a significant health problem worldwide. For example, 20% of senior adults in Western countries are considered “orally disabled,” and tooth loss is associated with a compromised quality of life, manifesting as difficulties in chewing and speaking, pain or alterations in other oral sensations such as stereognosis and proprioception, as well as impaired memory, cognitive and emotional functions (for reviews see Feine and Carlsson, 2003; Crocombe et al., 2009; Avivi-Arber et al., 2011; Trulsson et al., 2012; Sessle et al., 2013; Klineberg et al., 2014; Cerutti-Kopplin et al., 2016). Since life expectancy is progressively increasing in most populations, tooth loss and the associated impairments represent an increasing societal burden (Avivi-Arber et al., 2011; Trulsson et al., 2012). However, these effects of tooth loss vary substantially across individuals (Haraldson et al., 1988; Zarb et al., 2013; Carr and Brown, 2015), both in terms of the type and severity of the outcomes, and also in the rate and quality of recovery. This suggests that while these traits are impacted by variable environmental factors, they may also be under genetic influences which are still unknown (Mishra et al., 2007; Seltzer and Mogil, 2008; Katz and Seltzer, 2009; Missitzi et al., 2013). Finding ways to prevent or treat tooth loss-induced impairments depends on knowledge of the involved genetic, cellular, molecular, structural, and functional brain mechanisms, which is currently largely lacking.

Structural magnetic resonance imaging (sMRI) as well as functional MRI (fMRI) have been used to study how the brain responds to changes in orofacial functions. For example, human studies have revealed that altered dentitional states including tooth loss and their restoration are accompanied by widespread structural and functional brain changes in regions involved in processing and controlling sensory, motor, cognitive and emotional functions (Yan et al., 2008; Ono et al., 2010; Luraschi et al., 2013; Ohkubo et al., 2013; Shoi et al., 2014). In addition, such changes also occur following training and learning of oral motor skills, as well as in chronic orofacial pain conditions (Momose et al., 1997; Onozuka et al., 2002; Jiang et al., 2010, 2015; Arima et al., 2011; Gerstner et al., 2011; Gustin et al., 2011; Moayedi et al., 2011; Weissman-Fogel et al., 2011; Desouza et al., 2013). However, the cellular, molecular, and genetic mechanisms underlying these structural and functional changes are unclear but can be elucidated by utilizing brain imaging techniques in animals along with other invasive techniques such as electrophysiology and immunohistochemistry.

We and others have already shown that tooth extraction in rodents can also induce functional and structural changes in both glial and neuronal cells within brain regions involved in processing orofacial sensory and motor functions as well as cognitive and emotional behaviors (Avivi-Arber et al., 2014, 2015; Varathan et al., 2014; Chen et al., 2015; Watase et al., 2016). High resolution sMRI in rodents can provide an excellent readout of anatomical brain changes in mice following nerve injury, housing in an enriched environment, or maze training and such changes are also associated with cellular and molecular changes (Seminowicz et al., 2009; Lerch et al., 2011b; Cahill et al., 2015; Scholz et al., 2015a). However, it is still unclear whether sMRI can be utilized in mice to reveal volumetric regional brain changes following tooth extraction, whether it can detect volumetric regional brain differences in mice of different genetic background, and whether it can be utilized as a phenotyping method to identify genetic sources for inter-individual differences in brain plasticity following tooth loss.

As a first step, here our aim was to determine whether sMRI is sensitive to detect quantifiable volumetric differences in the brain of mice of different genetic background that received tooth extraction or sham operation. We randomly selected 7 strains, comprising A/J (‘A’) and C57BL/6J (‘B’) and 5 of the 23 AXB-BXA strains that were produced from the A and B parental mice by recombinations and inbreeding (Marshall et al., 1992; Lu et al., 1994; Sampson et al., 1998; Seltzer et al., 2001; Bennett et al., 2003). This panel of 25 genetically unique inbred strains has already been genetically mapped and has been widely used for mapping chromosomal intervals throughout the genome that harbor candidate genes controlling various phenotypes including spontaneous and stimulus-evoked neuropathic pain following orofacial and lumbar nerve injuries (Seltzer et al., 2001; Zhang S. H. et al., 2006; Nissenbaum et al., 2010; Mashregi et al., 2011; Soleimannejad et al., 2012).

## Methods

All experimental procedures were approved by the University of Toronto Animal Care Committee, in accordance with the Canadian Council on Animal Care Guidelines and the regulations of The Ontario Animals for Research Act (R.S.O. 1990). All experimental procedures (i.e., tooth extraction and sham operation, perfusions and preparation for the sMRI) were completed by the same investigator (LAA), adhering to the same standard protocols to ensure consistency. The sMRI data were analyzed in a blinded manner by one investigator (MF). Although, single housing might induce stress that could affect treatment outcome, control mice that received the sham treatment as well as mice receiving tooth extraction were single-housed to minimize social effects that might mask treatment effects (Devor et al., 2007; Seminowicz et al., 2009). Mice were housed in the same temperature and humidity-controlled environmental conditions, maintained at a 12-h light/dark cycle (lights switched on at 07:00 and off at 19:00 h). Starting at 2 weeks prior to the intraoral manipulation, all mice received a diet of mashed chow and water *ad libitum* to avoid discomfort from biting on a hard chow and to ensure adequate food and drink intake.

### Study groups

We utilized young adult (11–18 weeks) female mice of the following 5 recombinant inbred AXB-BXA strains: AXB1-PgnJ (termed ‘AXB1’; 5 extraction, 3 sham), AXB24-PgnJ (termed ‘AXB1’; 5 extraction, 3 sham), AXB4-PgnJ (termed ‘AXB4’; 3 extraction, 3 sham), BXA24-PgnJ (termed ‘BXA24’; 12 extraction, 9 sham;), and BXA14-PgnJ (termed ‘BXA14’; 7 extraction, 3 sham). These strains were randomly selected from the 23 AXB-BXA strain panel. In addition, we used 5 A (3 extraction, 2 sham) and 9 B mice (5 extraction, 4 sham). Breeding nuclei of all strains were originally purchased from Jax Labs (Bar Arbor, MI) and multiplied on demand in our animal facility. We used in this study female mice because the incidence of postoperative orofacial neuropathies is higher in women than in men (Macfarlane, 2014). A recent meta-analysis study suggests that utilization of female mice in studies such as the present does not require monitoring of the estrous cycle (Prendergast et al., 2014).

Mice were randomly allocated into Sham and Extraction groups and experiments were performed in a random sequence to reduce potential testing bias. For each strain, mice of the same age were assigned to undergo extraction or sham operation. Under general anesthesia, we extracted 3 right maxillary molar teeth in mice of the Extraction group (*n* = 39) (see procedure below), and mice in the Sham group (*n* = 28) received the same operation but without actual tooth extraction. Mice were monitored daily to assess food consumption, general behavior, and any postoperative complications such as bleeding or inflammation. Body weight was checked regularly to ensure a continuous gain of body weight. Mice demonstrated a slower rate of weight gain per day during the first 3–5 postoperative days but then resumed normal gain of body weight (0.5–1 g/d).

Consistent with our previously documented electro-physiological and immunohistochemical findings in rats and mice, demonstrating that changes in pain behavior and neuronal responses in the orofacial primary sensorimotor cortex are apparent on days 7–28 following orofacial injury (Zhang S. H. et al., 2006; Avivi-Arber et al., 2010a, 2015; Mashregi et al., 2011; Soleimannejad et al., 2012; Varathan et al., 2014; Hayashi et al., 2015), mice in the present study were killed humanely 21 days postoperatively. All mice were fixation-perfused transcardially and the brains were imaged as described below. Sixteen mice had to be excluded for technical reasons. The numbers of mice per strain listed above are the net included in the analysis.

### Molar tooth extraction and sham operation

Extraction and sham operations were carried out under general anesthesia (Isoflurane), using standard aseptic surgical conditions. Pulse oximeter monitoring verified that the heart rate and oxygen saturation levels were within the physiological range (i.e., 333–430 beats/min, 90–100% O_2_). A feedback-controlled heating pad maintained the mouse core temperature at 37–37.5°C. The mouth was kept open by pulling down the 2 mandibular incisors with a rubber band. In the Extraction group the 3 right maxillary molar teeth were luxated (Avivi-Arber et al., 2010b, 2015). Sham mice had the same general anesthesia and mouth opening but no actual tooth extraction. These procedures took up to 30 min. No analgesics or anti-inflammatory medications were administered postoperatively because of their possible confounding effect on the sMRI data.

### Structural magnetic resonance imaging

sMRI scanning and data analysis followed previously published standard protocols (Lerch et al., 2011a,b; Cahill et al., 2012) and are described below only briefly.

#### Sample preparation

Under general anesthesia (Ketamine HCl, 150 mg/kg and Xylazine, 10 mg/kg; i.p.) mice were perfused on postoperative day 21 with 30 ml mixture of 0.1 M phosphate-buffered saline (PBS), 10 U/mL Heparin and 2 mM ProHance® (an sMRI contrast enhancing agent), followed by perfusion of 30 ml 4% paraformaldehyde (PFA) in PBS and 2 mM ProHance®. The maxilla and skull containing the brain were post-fixed in 4% PFA and 2 mM ProHance® at 4°C for 12 h, then transferred to 4% PBS, 0.02% sodium azide and 2 mM ProHance® and stored at 4°C until scanned.

#### sMRI acquisition

A multi-channel 7.0 Tesla, 40 cm diameter bore magnet MRI scanner (Varian Inc. Palo Alto, CA) was used to acquire images of mouse brains. Brains were intact in their skulls and placed in Fluorinert, and 16 samples were scanned at one time in a 16-coil solenoid array. Parameters used were: a T2-weighted 3D fast spin-echo sequence, with TR = 2000 ms, echo train length = 6, TEeff = 42 ms, field-of-view = 25 × 28 × 14 mm, matrix size = 450 × 504 × 250, and voxel size = 56 × 56 × 56 μm (Lerch et al., 2011b; Cahill et al., 2012).

#### sMRI analysis

In order to visualize and compare changes across mice, an automated image registration-based approach was used to align all brains and create a consensus average (i.e., “atlas”). Image registration involved linear alignment of all images through a series of rotations, translations, scales, and shears. This was followed by locally deforming each scan through an iterative non-linear alignment procedure, bringing all scans into exact correspondence in an unbiased fashion. This registration was assumed to bring all homologous anatomical points into alignment. Next, the total brain volume was calculated for every mouse.

Our analytic approach to assess the volume of discrete brain regions between tooth extraction and sham operation was the following: First, we calculated the group mean volume of known neuroanatomical brain regions. For this purpose, a segmented atlas dividing the brain into 160 separate regions was aligned onto the study-population-specific atlas (Dorr et al., 2008; Ullmann et al., 2013; Steadman et al., 2014). Then, a deformation field was calculated for each mouse that determined how much the individual mouse's anatomy had to be transformed to fit the final atlas space, also facilitating the assessment of the degree to which this deformation field differed across mice in the study (Nieman et al., 2006; Lerch et al., 2011b). The determinant of each deformation field, known as Jacobian Coefficient (JC), was calculated for each voxel in the brain of every mouse. This coefficient is a measure of the deformation of each voxel with respect to the atlas image. It can be thought of as the amount by which the volume of that voxel had to be multiplied to reach the consensus average. Thus, a JC = 1 indicates no change, while JC > 1 signifies expansion and < 1 denotes shrinkage of that voxel volume with respect to the volume of the same voxel in the atlas image. The resulting atlas was then used in conjunction with the JCs (multiplied by an appropriate scaling factor) to calculate volumes for each region in the brain and for the brains of all mice in the study. The regional volumetric values of the right and left sides of the brains were averaged. Since we used a genetically heterogeneous population of mice from 7 strains that significantly differed in their mean total brain volumes (A: 391 ± 2; B: 472 ± 3; AXB1: 417 ± 8; AXB24: 398 ± 5; AXB4: 384 ± 8; BXA14: 397 ± 8; BXA24: 461 ± 4 mm^3^; *p* < 0.0005), the JC values used in this analysis were normalized for every mouse with respect to its overall brain volume. This approach allowed for examination of normalized regional brain volumes and comparison of anatomical differences across mice receiving tooth extraction or sham operation.

To test whether sMRI can detect significant differences between mice of different genetic background we utilized two strains, the BXA14 (*N* = 7 Extraction, 3 Sham) and BXA24 (*N* = 12 Extraction, 9 Sham) strains since they had the largest numbers of mice per group, they significantly differed in their total brain volume (*P* < 0.0001), and previous studies have shown they have contrasting tactile hypersensitivity following orofacial and lumbar nerve injuries (Seltzer et al., 2001; Zhang S. H. et al., 2006; Nissenbaum et al., 2010; Mashregi et al., 2011; Soleimannejad et al., 2012).

#### Statistical analysis

Significance of the treatment effect in the regional brain volume analysis was carried out by analysis of variance (ANOVA). Resulting probabilities were adjusted for multiple comparisons with the False Discovery Rate (FDR) set at 1%. For the voxel-wise analysis, we performed a two-sample *t*-test to compare significant voxel-wise differences between tooth extraction and sham operation. The *p*-values were adjusted by FDR at 1, 5, and 10%. Thereafter, these voxels were annotated by their neuroanatomical region (Genovese et al., 2002). The t-statistic values of the voxel-wise analysis were then used for graphically displaying brain statistical maps of treatment effects in all mice (i.e., regardless of their genetic differences) and separately again when treatment effect was compared between mice of the BXA14 and BXA24 strains.

## Results

### Anatomical differences between mice receiving tooth extraction vs. sham operation

#### Regional volumetric analysis

Normalized volumetric sMRI data analysis of 160 brain regions revealed 34 brain regions that showed significant bilateral volumetric differences (FDR correction at 5 and 10%; none at 1%) between mice receiving tooth extraction and those receiving sham operation (Table 1). We found significantly decreased gray matter volumes in several forebrain regions, including the primary somatosensory (S1), primary motor (M1), and the cingulate cortices in mice receiving tooth extraction as compared with sham-operated mice. Decreased volume was also observed in components of the basal ganglia (striatum, globus pallidus, and nucleus accumbens). Also notably, several temporal limbic forebrain regions, including the amygdala, and subcortical sensory and motor nuclei showed significantly increased gray matter volumes following tooth extraction as compared with sham operation.

**Table 1:** **Regional volumes (mm^**3**^) (Mean ± SEM) and % difference in the means of the normalized volumes between mice receiving tooth extraction and those receiving sham operation**.

**Brain region**	**Volume (mm^3^) (Mean + SEM)**		**Effect of extraction vs. sham**	**% difference from sham (normalized)**	***q-*****values**
	**Extract**	**Sham**			
**FOREBRAIN CORTEX**					
Lateral orbital cortex	2.86 ± 0.04	3.00 ± 0.05	Shrank	−2.86	0.083
Frontal association cortex	6.37 ± 0.11	6.71 ± 0.11	Shrank	−3.48	0.079
Primary motor cortex	6.50 ± 0.09	6.69 ± 0.09	Shrank	−2.12	0.076
Primary somatosensory cortex	4.58 ± 0.06	4.76 ± 0.06	Shrank	−2.22	0.057
Cingulate cortex (area 24a)	1.67 ± 0.02	1.74 ± 0.02	Shrank	−2.90	0.075
Cingulate cortex (area 25)	0.42 ± 0.01	0.45 ± 0.01	Shrank	−3.24	0.031
Cingulate cortex (area 32)	2.22 ± 0.04	2.34 ± 0.03	Shrank	−3.86	0.081
Medial entorhinal cortex	0.69 ± 0.01	0.68 ± 0.01	Expanded	2.91	0.037
Posteromedial cortical amygdalar area	1.18 ± 0.02	1.15 ± 0.02	Expanded	3.74	0.031
Amygdalopiriform transition area	23.00 ± 0.26	22.30 ± 0.27	Expanded	3.16	0.079
Posterolateral cortical amygdalar area	1.18 ± 0.02	1.15 ± 0.02	Expanded	2.24	0.086
Caudomedial entorhinal cortex	4.85 ± 0.09	4.77 ± 0.09	Expanded	3.20	0.030
Ventral intermediate entorhinal cortex	0.99 ± 0.02	0.98 ± 0.02	Expanded	3.03	0.065
**CEREBELLUM**					
Cerebellar lobule10 white matter	0.09 ± 0.00	0.08 ± 0.00	Expanded	7.08	0.031
Cerebellar lobule10 nodulus	1.50 ± 0.03	1.44 ± 0.03	Expanded	5.76	0.054
Cerebellar peduncle inferior	0.86 ± 0.01	0.84 ± 0.01	Expanded	3.75	0.054
Cerebellar peduncle (middle)	1.25 ± 0.02	1.23 ± 0.02	Expanded	3.22	0.085
**FOREBRAIN SUBCORTEX**					
Mammillary bodies	0.51 ± 0.01	0.49 ± 0.01	Expanded	5.82	0.031
Claustrum	0.30 ± 0.01	0.32 ± 0.01	Shrank	−5.32	0.020
Claustrum ventral part	0.51 ± 0.01	0.54 ± 0.01	Shrank	−2.48	0.044
Dorsal nucleus of the endopiriform	1.37 ± 0.02	1.43 ± 0.03	Shrank	−2.72	0.030
Striatum	19.94 ± 0.33	20.80 ± 0.43	Shrank	−2.35	0.076
Globus Pallidus	2.65 ± 0.05	2.75 ± 0.06	Shrank	−2.14	0.065
Nucleus accumbens	3.87 ± 0.06	4.03 ± 0.06	Shrank	−2.24	0.054
Fimbria	3.14 ± 0.06	3.28 ± 0.07	Shrank	−2.46	0.061
Bed nucleus of stria terminalis	1.12 ± 0.02	1.18 ± 0.03	Shrank	−3.18	0.036
**TRACTS**					
Mammilothalamic tract	0.24 ± 0.00	0.25 ± 0.00	Shrank	−2.18	0.077
Anterior commissure pars posterior	0.40 ± 0.01	0.42 ± 0.01	Shrank	−2.38	0.057
**BRAINSTEM**					
Periaqueductal gray	3.55 ± 0.07	3.67 ± 0.07	Shrank	−1.85	0.100
Pons	15.94 ± 0.28	15.87 ± 0.30	Expanded	2.08	0.061
Pontine nucleus	0.72 ± 0.01	0.69 ± 0.02	Expanded	5.65	0.061
Cuneate nucleus	0.25 ± 0.00	0.23 ± 0.00	Expanded	8.91	0.030
Superior olivary complex	0.79 ± 0.01	0.78 ± 0.01	Expanded	3.80	0.031
Medulla	26.25 ± 0.42	25.68 ± 0.42	Expanded	4.00	0.030

*Negative values denote regions where volumes were smaller in mice receiving tooth extraction (“Shrank”). Positive values denote regions where volumes were larger in mice receiving tooth extraction (“Expanded”). q-values (<0.1) represent FDR-adjusted p-values*.

#### Voxel-wise analysis

**Figure 1:**
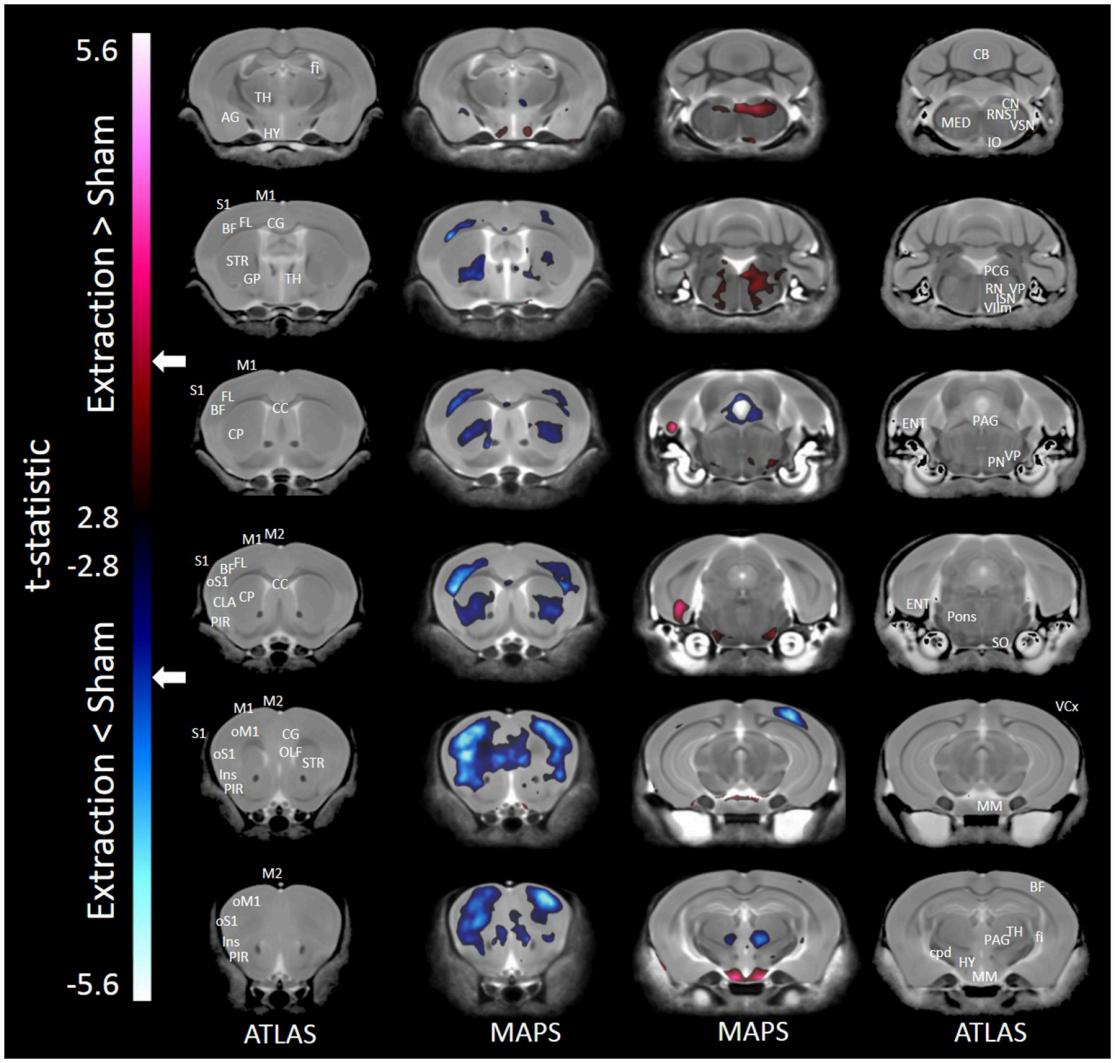
**Representative color-coded t-statistic maps (when all mouse strains were included) superimposed on high-resolution sMRI coronal slices of the mouse brain**. Images of coronal slices in the 1st and 4th columns show anatomical annotations. The coronal slices in the 2nd and 3rd columns are presented in a rostrocaudal order from top-left to bottom-right. Maps show normalized voxel-wise volumetric differences between mice receiving tooth extraction and those receiving sham operation. Red indicate regions that have larger voxel volumes in mice receiving tooth extraction than in mice receiving sham operation, whereas those in blue indicate regions that have smaller voxel volumes in mice receiving tooth extraction. All t-statistics shown are significant at a 10% FDR. Peaks associated with t-statistic values < −3.53 or >3.53 are significant at FDR = 5% (*t* = 3.53 and *t* = −3.53 are marked as white arrows on the color palettes). No voxel differences were significant at a 1% FDR. BF, barrel field; CC, corpus callosum; CB, cerebellum; CG, cingulate cortex; CLA, claustrum; CP, caudate putamen; cpd, cerebral peduncle; ENT, entorhinal cortex; fi, fimbria; FL, forelimb; GP, globus pallidus; HY, hypothalamus; Ins, insular cortex; IO, inferior olivary complex; ISN, inferior salivary nucleus; M1, primary motor cortex; M2, secondary motor cortex; MED, medulla; MM, mammillary nucleus; OLF, olfactory area; oM1, orofacial primary motor cortex; oS1, orofacial primary somatosensory cortex; PAG, periaqueductal gray; PCG, pontine central gray; PIR, piriform area; PN, pontine nuclei; RN, reticular nuclei; S1, primary somatosensory cortex; SO, superior olivary complex; ST, solitary tract nucleus; STR, striatum; TH, thalamus; VCx, visual cortex; VIIm, facial cranial nerve motor; VP, trigeminal principal nucleus; VSN, trigeminal spinal tract nucleus.

Results of the voxel-wise comparison between mice receiving tooth extraction and those receiving sham operation are shown in Figure 1. Compared to sham operation, tooth extraction was associated with significantly decreased volume in many brain regions, including orofacial sensorimotor processing regions such as the S1, M1, and insular cortices, the basal ganglia (i.e., caudate, globus pallidus, and nucleus accumbens), paraventricular nucleus of the thalamus and trigeminal motor nucleus. Regions that showed significantly increased volume following tooth extraction included the entorhinal cortex, facial nerve and nucleus, cuneate nucleus, hypothalamus, inferior olivary complex, periaqueductal gray, pons, solitary tract nucleus, and trigeminal spinal tract nucleus. Notably, in no brain region were the observed effects of regional-based and voxel-wise analyses contradictory.

### Anatomical differences between mice of different genetic backgrounds

#### Voxel-wise analysis

**Figure 2:**
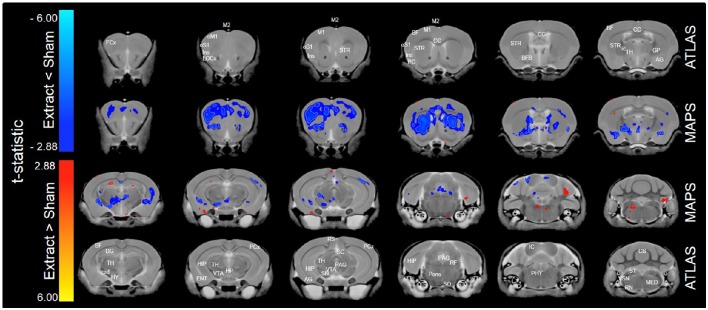
**Representative color-coded t-statistic maps (when only the BXA14 and BXA24 strains were included) superimposed on high-resolution sMRI coronal slices of the mouse brain**. Images of coronal slices in the 1st and 4th rows show anatomical annotations. The coronal slices in the 2nd and 3rd rows are presented in a rostrocaudal order from top-left to bottom-right. Maps show normalized voxel-wise volumetric differences between mice receiving tooth extraction and those receiving sham operation. Red indicate regions that have larger voxel volumes in mice receiving tooth extraction than in mice receiving sham operation, whereas blue indicate regions that have smaller voxel volumes in mice receiving tooth extraction. All t-statistics shown are significant at a 10% FDR. White contour lines delineate regions where the statistical maps are significant at 5% FDR. No voxel differences were significant at a 1% FDR. AG, amygdalar cortex; BF, barrel field; BFB, basal forebrain; CC, corpus callosum; CB, cerebellum; cpd, cerebral peduncle; DG, dentate gyrus; ENT, entorhinal cortex; FCx, frontal cortex; GP, globus pallidus; HIP, hippocampus; HP, hypothalamic nucleus; HY, hypothalamus; ic, internal capsule; IC, inferior colliculus; Ins, insular cortex; LOCx, lateral orbital cortex; M1, primary motor cortex; M2, secondary motor cortex; MED, medulla; oM1, orofacial primary motor cortex; oS1, orofacial primary somatosensory cortex; PAG, periaqueductal gray; PCx, Parietal cortex; PC, Piriform cortex; PHY, perihypoglossal nuclei; RF, reticular formation; RN, reticular nucleus; RS, retrosplenial; SC, superior colliculus motor related; SN, substantia nigra; SO, superior olivary complex; ST, solitary tract nucleus; STR, striatum; TH, thalamus; VSN, trigeminal spinal nucleus; VTA, ventral tegmental area.

**Figure 3:**
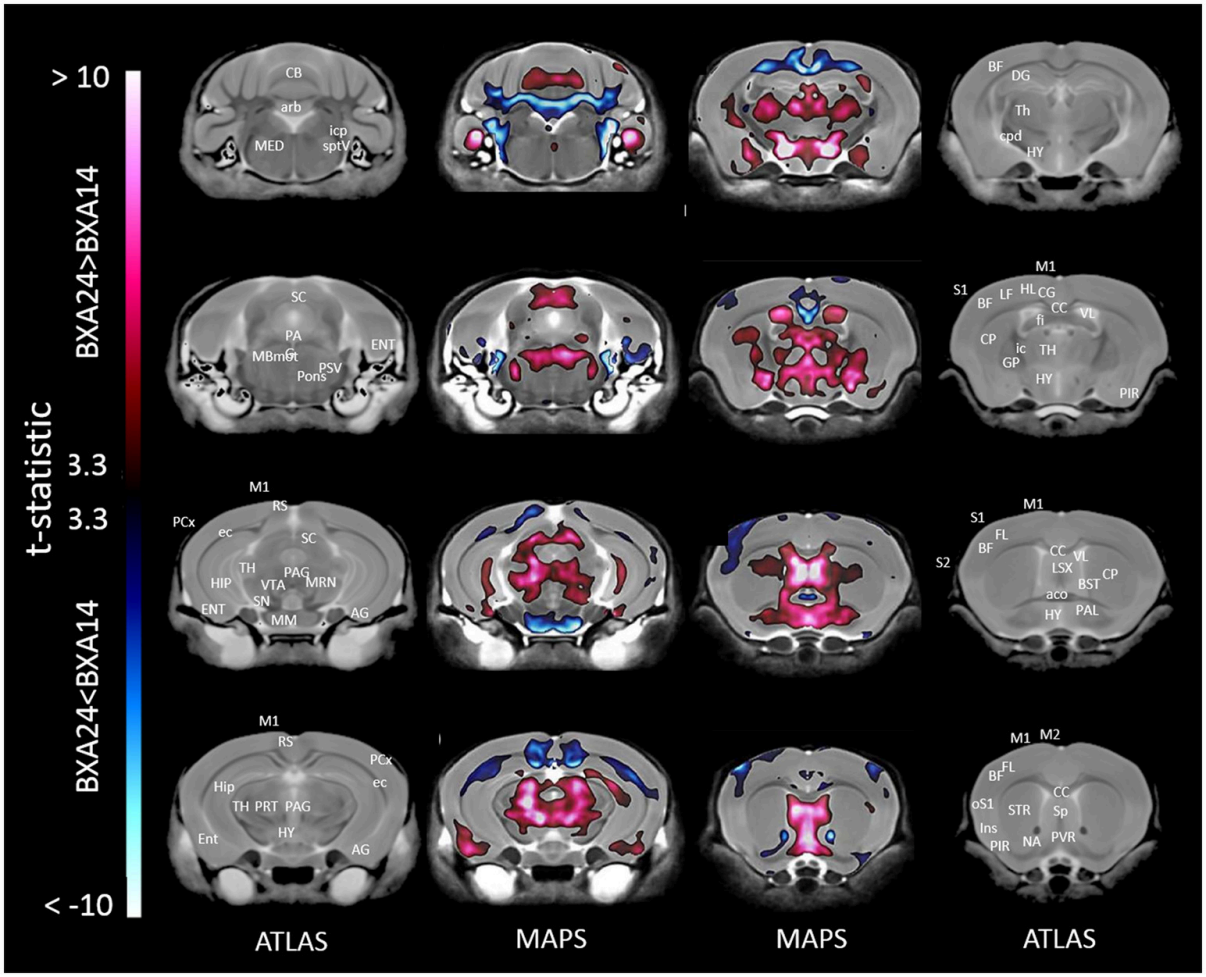
**Representative color-coded t-statistic maps superimposed on high-resolution sMRI coronal slices of the mouse brain**. Images of coronal slices in the 1st and 4th columns show anatomical annotations. The coronal slices in the 2nd and 3rd columns are presented in a rostrocaudal order from bottom-right to top-left. Maps show normalized voxel-wise volumetric differences between BXA14 and BXA24 mice. Red indicate regions that have larger voxel volumes in BXA24 mice than in BXA14 mice, whereas those in blue indicate regions that have larger voxel volumes in BXA14 mice. All t-statistic values shown are significant at 1% FDR. Abbreviations: aco, anterior commissure; AG, amygdalar cortex; arb, arbor vitae; BF, barrel field; BST, bed nucleus of stria terminalis; CB, cerebellum; CC, corpus callosum; CG, cingulate cortex; CP, caudate putamen; cpd, cerebral peduncle; DG, dentate gyrus; ec, external capsule; ENT, entorhinal cortex; fi, fimbria; FL, forelimb; HIP, hippocampus; HL, hindlimb; HY, hypothalamus; ic, internal capsule; icp, inferior cerebellar peduncle; Ins, insular cortex; LSX, lateral septal complex; M1, primary motor cortex; M2, secondary motor cortex; MBmot, midbrain, motor related; MED, medulla; MM, mammillary nucleus; MRN, midbrain reticular nucleus; NA, nucleus accumbens; oS1, orofacial primary somatosensory cortex; PAG, periaqueductal gray; PAL, pallidum; PCx, posterior parietal association area; PIR, piriform area; PRT, pretectal region; PSV, principal sensory nucleus of the trigeminal; PVR, periventricular area; RS, retrosplenial; S1, primary somatosensory cortex; S2, secondary somatosensory cortex; SC, superior colliculus; SN, substantia nigra; Sp, septal nuclei; sptV, spinal tract of the trigeminal nerve; STR, striatum; TH, thalamus; VL, lateral ventricle; VTA, ventral tegmental area.

When only mice of the BXA14 and BXA24 were included in the data analysis, we found that the normalized voxel-wise volumetric differences between mice receiving tooth extraction and those receiving sham operation (Figure 2) were similar but more significant than those obtained when all seven strains were included in the data analysis (Figure 1). In addition, voxel-wise comparison between the BXA14 mice and BXA24 mice (Figure 3) showed that irrespective of the dental manipulation, many normalized brain regions, including the periaqueductal gray and thalamus, were significantly larger (FDR = 1%) in BXA24 mice than in BXA14 mice. Other brain regions such as the M1, S1, nucleus accumbens, arbor vita of the cerebellum, and corpus callosum, were significantly larger (FDR = 1%) in BXA14 than in BXA24 mice.

## Discussion

This is the first study to show that *post-mortem* sMRI is a sensitive method capable of detecting significant differences in the volume of brain regions between mice of different genetic background and between mice that had tooth extraction vs. sham operation. The study provides novel findings that tooth loss in a genetically heterogeneous population of mice leads to widespread bilateral gray matter changes. Specifically, we found that tooth extraction leads to: (1) reduced gray matter volume in several forebrain regions including the sensorimotor cortex, insula, cingulate cortex, and basal ganglia; (2) increased gray matter volume in several brainstem sensory and motor nuclei, and in the cerebellum; (3) increased gray matter volume in several cognitive and limbic brain regions, including the anterior association cortex, nucleus accumbens, entorhinal cortex, mammillary bodies, and the amygdala. Together, these findings highlight the tremendous impact of tooth loss on brain structures across sensory, motor, and limbic systems.

### Effects of tooth loss on brain anatomy

#### Tooth loss related plasticity in sensory and motor brain regions

One of the main findings of this study was that tooth loss leads to gray matter decreases in the basal ganglia, and in the S1 and M1 cortices. The shrinkage of M1 and S1 is consistent with previous findings of decreased jaw and tongue motor representations and decreased excitability of orofacial M1 and S1 following molar tooth extraction in rodents (Avivi-Arber et al., 2015; Hayashi et al., 2015). Such changes may reflect the documented adaptive or maladaptative processes induced by the altered somatosensory inputs as a result of the missing teeth, injury to gingival, periodontal and pulpal nerves, and/or by compensatory sensorimotor functions caused by the loss of three major food-grinding dental elements (for review see Avivi-Arber et al., 2011; Sessle et al., 2013).

We also found that tooth extraction induced volumetric expansion in several brainstem regions involved in sensory and motor functions. These include the trigeminal motor nucleus, facial nucleus and nerve, the trigeminal sensory and solitary tract nuclei, pons, superior and inferior olivary complexes, and the cuneate nucleus. These novel findings, of changes in brainstem sensory and motor nuclei that are related to orofacial sensory and motor functions, are consistent with electrophysiological studies in rats. In these studies we and others have shown that acute or chronic dental pulpitis or trigeminal nerve damage induced functional neuroplasticity reflecting an increased glutamate-mediated excitability (“*central sensitization*”) of nociceptive neurons within the brainstem trigeminal subnucleus caudalis that process orofacial nociceptive afferent inputs (for review see Sessle, 2011), and increased neuronal activity in the trigeminal motor nucleus (Sunakawa et al., 1999; Mostafeezur et al., 2014). Central sensitization following dental manipulations has also been documented in the rodent somatosensory thalamus (Park et al., 2006; Zhang S. et al., 2006; Kaneko et al., 2011). Central sensitization contributes to increased pain sensitivity (allodynia and hyperalgesia) and extraterritorial spread or referral of pain hypersensitivity that characterize many acute and chronic pain conditions (for review, see Sessle, 2011).

In addition, we showed here that tooth extraction was also associated with reduced gray matter volume in the frontal association cortex, an area that is mainly responsible for complex processes involving inputs to the cerebral cortex and the generation of behaviors including motor planning, working memory, and problem solving (Purves et al., 2012).

Compared to sham-operated mice, mice that underwent tooth extraction also had less gray matter volume in the basal ganglia, including the striatum, globus pallidus, and nucleus accumbens—regions related to motor processing and motivation. Specifically, nucleus accumbens is involved in reward processing and motor recovery after injury, and also shows abnormal response to noxious stimuli in subjects suffering from various chronic pain conditions (Pliakas et al., 2001; Perrotti et al., 2008; Baliki et al., 2010; Gustin et al., 2011; Desouza et al., 2013; Ikeda et al., 2015; Sawada et al., 2015; Elman and Borsook, 2016).

We are unaware of comparable MRI studies in humans following tooth loss, however, decreased gray matter volume in the premotor cortex has been associated with decreased masticatory performance (Lin et al., 2016). Functional brain imaging studies have revealed that dental stimulation, chewing, and tooth clenching are associated with activation of several nodes of the sensorimotor network, including the S1, M1, premotor and supplementary motor cortices, insula, cerebellum, striatum, and thalamus (Momose et al., 1997; Onozuka et al., 2002; Ettlin et al., 2004; Miyamoto et al., 2006; Luraschi et al., 2013; Shoi et al., 2014; Jiang et al., 2015). Moreover, several of these studies have shown that the level of brain activity is related to the degree of biting force or dentitional state (i.e., whether it was fully dentate, partially- or completely edentate, or restored with dental implants). In addition, although tooth extraction is usually associated with a transient postoperative pain lasting up to 7 days, 0–3% of the patients undergoing tooth extraction develop chronic pain (Marbach and Raphael, 2000), and several fMRI studies have documented functional and structural changes in the brain of humans suffering from various chronic orofacial pain conditions. These studies identified structural abnormalities in brain regions such as the S1, M1, insular, and cingulate cortices (e.g., Nash et al., 2010; Gerstner et al., 2011; Gustin et al., 2011; Moayedi et al., 2011; Weissman-Fogel et al., 2011; Desouza et al., 2013; Youssef et al., 2014). Thus, the pattern of structural changes observed in the present study in mice, and those of other oral manipulations in humans, support the view that sMRI in mice can adequately detect quantitative brain volumetric changes following tooth loss, and that this method can serve as a research platform to better understand these changes so that treatments can be developed to maximize adaptation and minimize possible concomitant maladaptation following tooth loss.

#### Tooth loss-related changes in cognitive and emotional functions

Emerging evidence from human and animal studies have reported that tooth loss may be a contributing factor to cognitive and memory decline (for review see Klineberg et al., 2014; Palla, 2015; Cerutti-Kopplin et al., 2016). It is commonly known that tooth loss can induce intense emotional distress (Okoro et al., 2012; Wiener et al., 2015). In the present study, tooth loss was associated with volume loss in the frontal association cortex and nucleus accumbens. The frontal association cortex is involved in motor planning, working memory, and problem solving; whereas the nucleus accumbens has been implicated in the formation of emotional memories (e.g., pain, stress; Pliakas et al., 2001; Perrotti et al., 2008; Elman and Borsook, 2016). Volume loss in the frontal association cortex and nucleus accumbens in humans has been correlated with cognitive decline (de Jong et al., 2012). Moreover, the present study revealed that the mammillary bodies, which play a crucial role in memory consolidation (Vann and Aggleton, 2004), underwent significant expansion following tooth extraction. Tooth extraction was also associated with expansion of the amygdala and entorhinal cortex. These brain regions are part of the limbic system and were found to be enlarged in humans and animals subjected to fearful conditions (De Bellis et al., 2000; Lupien et al., 2009; van der Plas et al., 2010; Scholz et al., 2015b). Thus, the increased volume of these regions in the present study may be related to excessive fear or anxiety in response to the tooth loss.

### Effects of genetic differences on brain anatomy

We report here significant volumetric differences between the BXA14 and BXA24 strains for which we had sufficient numbers of mice per group to determine a statistically significant genetic effect. The total brain volume of the BXA14 mice was strikingly smaller than that of BXA24. This is consistent with a previous histological report of significant differences in total brain size across all 25 strains of the AXB-BXA strain panel (including BXA14 and BXA24) (Cutler Strom, 2002). Since in the present study regional volumes were normalized by total brain volume, the differences between BXA14 vs. BXA24 reflect true strain-dependent volumetric plasticity following tooth extraction.

Using histological preparations to measure the size of the cerebral ventricles it was reported that there are significant differences across strains of the AXB-BXA panel (Zygourakis and Rosen, 2003). But the present study is the first to use sMRI to compare 160 different regions of known structural and functional importance in the whole brain of two of the strains of this panel. We have shown that the two strains significantly differ in the normalized voxel-wise volumes of many gray and white matter regions. For example, the M1 and S1 cortices, nucleus accumbens, arbor vita of the cerebellum and corpus callosum were significantly larger in BXA14 than in BXA24 mice whereas the periaqueductal gray and thalamus showed the opposite effect. Since the non-genetic, environmental parameters of this study were identical for all mice, the strain-dependent differences in brain regional volume indicate a difference in the genetic control of this trait. As these mice were derived from the A and B strains by recombinations, these parental strains must also differ in the genetic control of regional brain volumes. This feature can be used to study the genetic underpinning controlling this trait by capitalizing on the availability of the genetic map of all 25 strains of this panel (Sampson et al., 1998). Knowing the regional volumes of all 25 strains for intact mice and those receiving the sham-operation or tooth extraction will enable mapping the chromosomal regions that harbor the genes controlling the brain regional volumes of naïve female mice and those controlling the volumetric changes caused by tooth extraction (http://www.genenetwork.org/home.html). The present findings are the first step in this direction by demonstrating that sMRI is sensitive enough to quantify differences in regional brain volumes of strains in the AXB-BXA panel.

### Possible mechanisms underlying tooth loss-induced volumetric brain changes

The mechanisms involved in changes in regional brain volume following intraoral injuries such as tooth extraction are not well known. Such volumetric alterations may reflect structural changes in glia, neurons, and blood vessels that result from changes in their number, function and/or volume (Zatorre et al., 2012), as well as volumetric changes caused by changes in the extracellular space. While there are many neurophysiological consequences of tooth loss, none of these can explain how they cause or relate to the different volumetric changes that we report here. The most obvious neurophysiological consequences of tooth loss are alterations in somatosensory (including nociceptive and proprioceptive) inputs from the lost teeth, injured gums and periodontal ligaments that are relayed via injured primary afferents, ascending tracts, and relay nuclei to multiple brain regions (Sessle, 2009; Avivi-Arber et al., 2011; Sessle et al., 2013). Somatosensory inputs from the tongue, jaw muscles, and temporomandibular joints may also be altered since tooth loss is associated with changes in masticatory patterns (Miehe et al., 1999; Klineberg and Jagger, 2004). Changes in the trigeminal brainstem sensory nuclei, thalamus, S1, M1 as well as in other higher order brain regions were documented by us previously (for review see Avivi-Arber et al., 2011; Sessle et al., 2013). For example, we have shown in rodents and humans that changes in somatosensory inputs or altered motor functions induced by intraoral manipulations, including tooth extraction, can result in short-term (days) and long-term (months) functional neuroplasticity in the orofacial S1 and M1 (Adachi et al., 2007; Avivi-Arber et al., 2010a, 2015; Awamleh et al., 2015; Pun et al., 2016). Tooth loss results in decreased motor representation of jaw and tongue muscles and decreased orofacial M1 excitability (Avivi-Arber et al., 2015). Moreover, acute dental stimulation, pulpectomy, or trigeminal nerve injury produces increased excitability of neurons within the ascending trigeminal somatosensory pathways including the trigeminal brainstem sensory nuclei, and decreased excitability in the orofacial sensorimotor cortex (e.g., Hu et al., 1986; Kwan et al., 1993; Adachi et al., 2007; Okada-Ogawa et al., 2009; Tsuboi et al., 2011; Chiang et al., 2012; Cao et al., 2013; Awamleh et al., 2015; Pun et al., 2016). We have also shown that these functional neuroplastic changes are dependent on the functional integrity of glial cells (Okada-Ogawa et al., 2009; Chiang et al., 2011, 2012; Tsuboi et al., 2011; Awamleh et al., 2015; Pun et al., 2016), and are associated with changes in the number and cytoarchitectural features of neurons and glia within the trigeminal brainstem sensory nuclei as well as the orofacial S1 and M1 (Okada-Ogawa et al., 2009; Tsuboi et al., 2011; Varathan et al., 2014; Avivi-Arber et al., 2015; Watase et al., 2016). However, as discussed above, none of these functional changes has ever been shown to causally drive the structural changes or *vice versa*. More research is needed to unravel the mechanisms underlying the sMRI plasticity observed in the current study and to link them to the documented neuronal and glial changes following intra-oral injuries.

## Conclusions and implications for future studies

The current study has demonstrated, for the first time, that high-resolution sMRI can be used for quantifying small volumetric brain differences between mice of different genetic background and between mice receiving tooth extraction vs. sham operation. The novel findings show that tooth loss is associated with widespread sMRI-defined structural changes in somatosensory, motor, cognitive, and limbic regions of the brain. The robust findings reported here were obtained from a genetically diverse population of mice of 7 different strains, thereby modeling the effect of tooth loss in heterogeneous cohorts of humans. The findings also show significant sMRI-defined structural differences between two strains (BXA14 and BXA24) of the AXB-BXA panel. Future studies using sMRI are warranted to phenotype brain structural plasticity in all 23 recombinant inbred strains of the AXB-BXA panel and their A and B parental strains, to map the murine genome for chromosomal regions harboring candidate genes controlling the volumetric brain changes induced by tooth loss, implementing the already documented high-resolution genetic map of this panel (Seltzer et al., 2001; Zhang S. H. et al., 2006; Zhang et al., 2014; Nissenbaum et al., 2010; Mashregi et al., 2011; Meloto et al., 2011; Soleimannejad et al., 2012; Seltzer, 2014). Additionally, the volumetric brain changes can be correlated with behavioral changes caused by tooth loss (e.g., altered cognitive behavior, orofacial mechanical sensitivity, patterns of mastication) as well as changes in gene expression and cellular, molecular, micro-functional, and micro-structural changes. Such studies can provide a powerful multidisciplinary approach to elucidate mechanisms underlying sMRI-defined macrostructural brain changes produced by tooth loss and other orofacial manipulations of clinical relevance in humans (Clarke et al., 2015). The findings of these studies have the potential to identify new targets for the prevention and treatment of maladaptive behaviors after tooth loss and other orofacial manipulations.

## Author contributions

Conceived the study: LA, ZS, BS, JL, MM, and KD. Designed the experiments: LA, ZS, BS, MF, and JL. Performed the experiments: LA, MF, and JL. Analyzed the data: LA, ZS, BS, MF, and JL. Wrote the paper: LA, ZS, BS, MF, JL, MM, and KD.

### Conflict of interest statement

The authors declare that the research was conducted in the absence of any commercial or financial relationships that could be construed as a potential conflict of interest.
